# Non-traumatic Medial-Sided Elbow Pain: A Comprehensive Review of Etiologies, Diagnostic Strategies, and Treatment Approaches

**DOI:** 10.7759/cureus.94701

**Published:** 2025-10-16

**Authors:** Nadhila Adani, Xarisa Azalia, Karina S Gani, Mitchel Mitchel, Erica Kholinne

**Affiliations:** 1 General Practice, YARSI University, Jakarta, IDN; 2 School of Medicine and Health Sciences, Atma Jaya Catholic University of Indonesia, Jakarta, IDN; 3 Orthopedics and Traumatology, Gatam Institute, Eka Hospital, Tangerang, IDN; 4 Orthopedics and Traumatology, Faculty of Medicine, Trisakti University, Jakarta, IDN

**Keywords:** medial elbow pain, medial epicondylitis, non-traumatic, sports, ulnar collateral ligament

## Abstract

Medial elbow pain is a rare and often underrecognized condition. In non-traumatic cases, such as medial epicondylitis (ME), ulnar collateral ligament (UCL) injury, cubital tunnel syndrome, snapping medial triceps, and posteromedial impingement, the clinical presentations are often similar, making diagnosis challenging. This narrative review aims to synthesize current evidence regarding the etiology, pathophysiology, diagnostic approaches, and treatment strategies for non-traumatic medial elbow pain. Relevant studies were evaluated to examine clinical assessments, imaging modalities, conservative management protocols, and surgical interventions associated with these conditions. Diagnosis is primarily guided by the appropriate history-taking, physical examination, or provocative test, and the application of imaging modalities, such as ultrasound (US) and magnetic resonance imaging (MRI), to achieve an accurate diagnosis. Although most non-traumatic medial elbow pain conditions can be managed with conservative treatment, surgical intervention may be considered in cases involving ulnar nerve involvement or when conservative therapy fails.

## Introduction and background

Medial elbow pain is a relatively uncommon condition, but it can be debilitating, particularly for athletes and individuals engaged in repetitive overhead activities. While the prevalence of conditions like medial epicondylitis (ME) is reported to be less than 1% in the general population [1], the impact of medial elbow pain on both athletic and occupational performance can be significant. This review explores a variety of conditions contributing to medial elbow pain, including but not limited to ME, ulnar collateral ligament (UCL) injuries, and snapping medial triceps. These conditions may be characterized by distinct pathophysiological mechanisms; for example, snapping medial triceps involves an abnormal movement of the triceps tendon over the medial epicondyle, and posteromedial impingement refers to the irritation of soft tissues in the posteromedial aspect of the elbow [2,3].

The aim of this literature review is to provide a comprehensive synthesis of the causes, diagnostic techniques, and management strategies for medial elbow pain. The review is organized by condition, diagnostic method, and treatment approach to offer a structured and practical guide for clinicians managing this challenging issue.

## Review

Methods

Study Design and Setting

This study was a narrative review based on a search of academic databases. The study conducted a comprehensive literature search in the PubMed and Google Scholar databases up to March 8, 2025, using keywords such as “medial elbow pain”, "elbow pain", "medial epicondylitis", “ulnar collateral ligament”, "diagnosis", and "treatment". The inclusion criteria specified studies published in English, peer-reviewed, and providing information on the etiology, diagnostic strategies, and treatment approaches for medial elbow pain. Eligible study designs included prospective and retrospective investigations, as well as case series and review articles. Exclusion criteria were studies without full-text availability, non-English publications, non-peer-reviewed works, expert opinions, and editorial articles.

**Figure 1:**
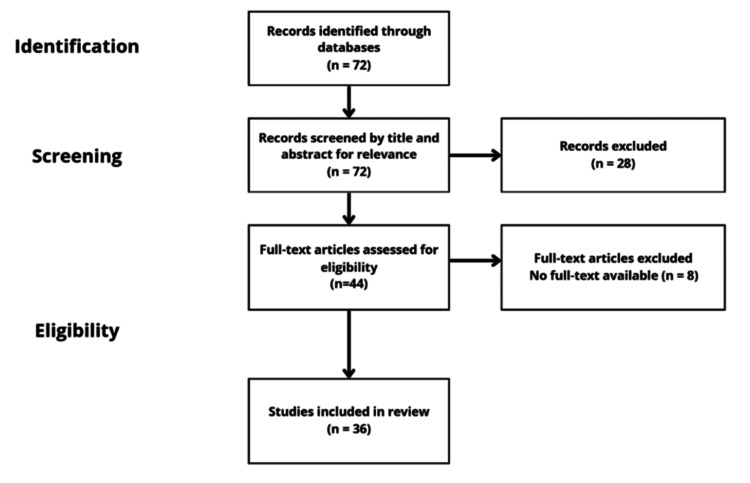
Study selection process

A total of 72 records were identified through database searches. After screening titles and abstracts, 72 records were advanced to full-text review. Of these, 44 articles were assessed for eligibility. Full-text review excluded eight articles for not meeting the inclusion criteria or no full text available. Finally, 36 studies were included in the review (Figure 1).

Medial Epicondylitis (ME)

ME, also known as golfer’s elbow, is most commonly seen in middle-aged individuals. It is linked to risk factors such as smoking, obesity, and repetitive activities or occupations that involve wrist flexion and forearm pronation [1]. The condition is characterized by tendinopathy of the flexor-pronator origin due to repetitive microtrauma [4].

Histopathologically, ME is marked by chronic tendinosis rather than acute inflammation. Repetitive overuse leads to microtears in the tendon, which in turn stimulate disorganized collagen remodeling, increased mucoid ground substance, decreased tensile strength, and the development of scar tissue and tendon thickening [5].

Clinical Evaluation

Patients typically maintain a full range of motion (ROM) but present with tenderness just distal to the ME and experience referred pain during activities that place strain on the flexor-pronator muscle origin [2]. Although this condition is commonly known as golfer's elbow, it can also affect individuals involved in tennis, weightlifting, swimming, or repetitive occupational tasks. Those who handle objects heavier than 5 kg for more than two hours daily or lift items over 10 kg more than 10 times per day are at increased risk of developing ME [6].

Physical examination may reveal localized tenderness approximately 5 to 10 mm distal and anterior to the ME, often accompanied by soft tissue swelling. Pain is typically aggravated by resisted wrist flexion, forearm pronation, or forceful gripping, and these movements may also demonstrate reduced strength compared to the contralateral side. Most patients retain normal passive and active ROM [7].

Diagnosis of ME is primarily clinical, supported by provocative tests. Passive stretch (supination with full extension of the elbow, wrist, and fingers) and resisted wrist flexion with pronation often reproduce pain [8]. The Polk test can further differentiate medial from lateral epicondylitis. During the test, the patient is seated with the elbow flexed at approximately 100° and the forearm in a supinated position. The examiner then instructs the patient to grasp and lift an object weighing around 2.5 kg. If the patient experiences pain at the medial epicondyle, the test is considered positive for ME. However, its diagnostic accuracy remains limited [8,9]. Overall, these tests are helpful but lack standardized validation, underscoring the importance of clinical judgment.

Imaging

Radiographs are typically normal in cases of ME, but are useful for excluding other potential pathologies. MRI provides superior soft tissue detail and remains the reference standard for diagnosis, particularly when evaluating concomitant pathologies such as UCL injury or osteochondral lesions. US offers a cost-effective and dynamic alternative, with reported sensitivity of 95% and specificity of 92%, though accuracy is operator-dependent [7,10,11].

In ME, US may reveal thickening and heterogeneous echotexture of the common flexor tendon (CFT), with hypoechoic or anechoic regions suggesting focal degeneration [12-14]. On MRI, hallmark findings include CFT thickening and increased T2 signal intensity with surrounding paratendinous edema [15,16]. In chronic cases, plain radiographs may show sclerotic changes or calcifications near the ME, especially in throwing athletes [10,17].

Conservative Treatment

Conservative treatment remains the cornerstone of initial management for ME and is typically successful in the majority of cases. First-line therapies include physical therapy focused on stretching and strengthening, cryotherapy, oral analgesics, and nonsteroidal anti-inflammatory drugs (NSAIDs) to reduce pain and inflammation [18-20]. Adjunctive modalities such as iontophoresis, acupuncture, and the use of orthotic bracing (e.g., counterforce straps or wrist splints) may provide additional symptomatic relief [2].

Injection-based therapies have gained popularity for patients who fail to respond to initial conservative measures. These include corticosteroid injections, platelet-rich plasma (PRP), autologous blood injections, and dry needling techniques. PRP and autologous blood injections have shown promise in promoting tendon healing through biological augmentation, though high-quality comparative data remain limited [19,20]. A retrospective study by Bohlen et al. found that leukocyte-rich PRP therapy significantly accelerates recovery in patients with ME (P < 0.01). The average time to achieve full ROM was significantly shorter with PRP (42.3 days) compared to surgical treatment (96.1 days). Similarly, the time to reach a pain-free status was reduced in the PRP group (56 days) versus the surgical group (108 days). Despite the faster recovery associated with PRP, the success rate was slightly higher in the surgical group (94%) compared to the PRP group (80%) [21].

Surgical Treatment

Surgery is considered for patients who have persistent symptoms after at least six months of conservative treatment. It is typically recommended when pain and limited function persist despite physical therapy, medications, and other nonoperative options [2]. The most common procedure is open debridement of the flexor-pronator origin, which may be combined with tendon repair if needed [7]. Another option is ME decortication, which involves removing a small amount of bone to improve blood flow and promote healing. If ulnar nerve irritation (neuritis) is present, ulnar nerve decompression or anterior transposition may be performed to relieve pressure on the nerve [2]. Arthroscopic debridement is a less invasive technique that has shown promise in laboratory studies, but there is limited evidence on its effectiveness in clinical practice. A retrospective study by do Nascimento and Claudio concluded that arthroscopic surgical treatment for ME of the elbow is a safe, effective approach that yields favorable clinical outcomes [22].

Postoperative protocols typically involve short-term immobilization followed by progressive mobilization and rehabilitation. Open procedures have shown an 80-85% success rate; however, the presence of ulnar neuropathy may negatively influence outcomes. A subset of patients may experience residual pain during high-demand activities [23,24].

UCL injury

**Figure 2:**
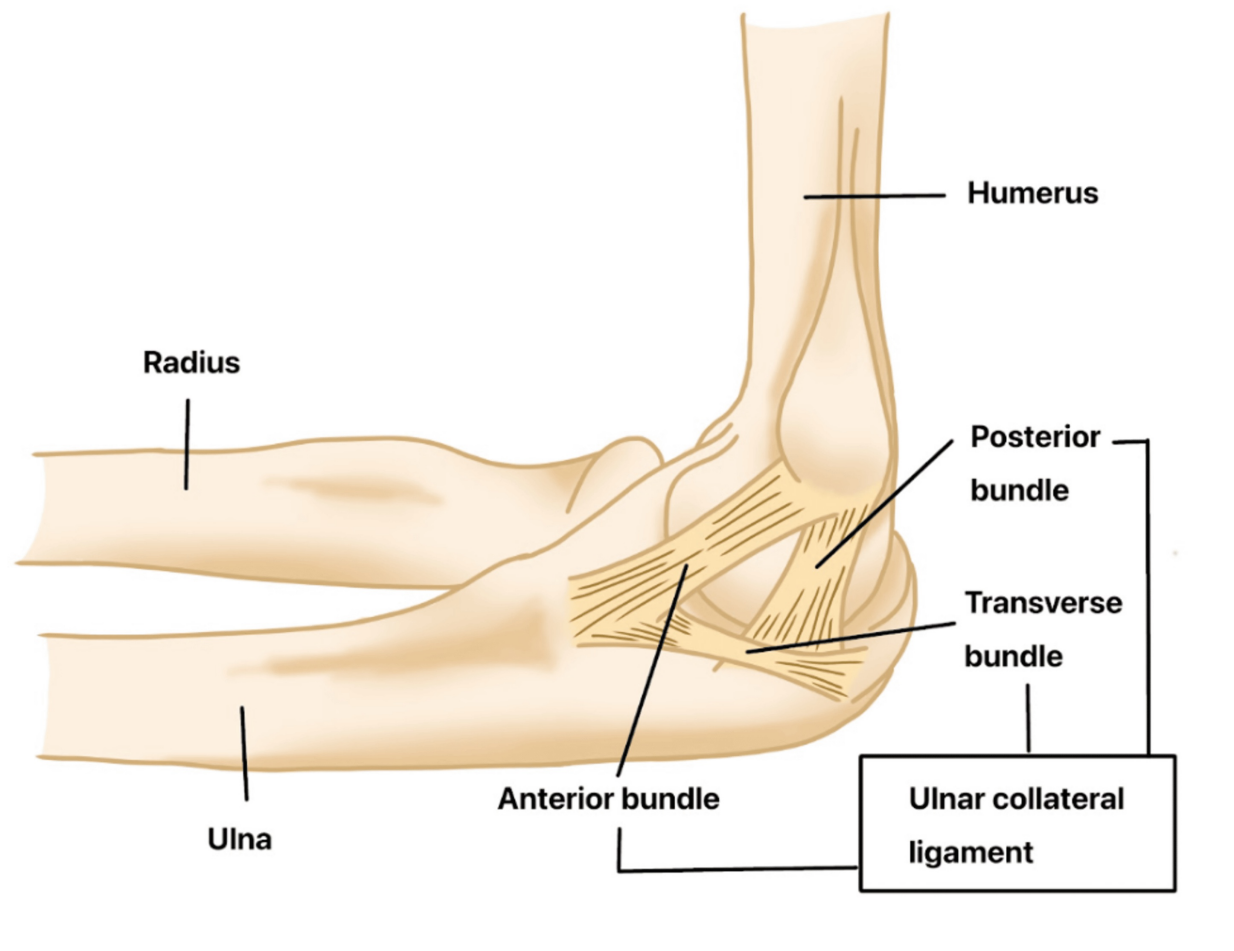
Anatomy of the ulnar collateral ligament (UCL) complex Image credit: The authors.

While ME is a common cause of medial elbow pain, another important differential diagnosis is UCL injury. The UCL is composed of three bundles: anterior, posterior, and transverse. The anterior bundle attaches broadly to the sublime tubercle and serves as the primary stabilizer of the elbow against valgus stress, making it the most important functional component of the UCL (Figure 2) [25]. UCL is a complex that consists of anterior, transverse, and posterior bundles. UCL injuries are prevalent in athletes, particularly baseball pitchers, but also occur in wrestlers and contact sports [25,26]. In throwing athletes, UCL injuries typically result from repetitive valgus stress during the throwing motion, gradually causing chronic degeneration of the ligament. This often presents with subtle symptoms, including reduced pitch velocity, prolonged warm-up time, or medial elbow discomfort following activity. Conversely, in contact athletes, UCL injuries are usually the result of an acute traumatic valgus load, leading to a sudden avulsion or rupture of an otherwise healthy ligament [27].

Clinical Evaluation

The typical presentation involves a gradual onset of medial elbow pain, particularly during the late cocking or acceleration phase of throwing. Some patients may also report a decline in throwing velocity and accuracy. In certain cases, a sudden “pop” may be felt, followed by an inability to continue throwing activities. Additionally, the disorders may lead to valgus elbow instability [28,29]. During physical examination, patients commonly exhibit tenderness on the medial aspect of the elbow, located just anterior and distal to the ME [2].

The moving valgus stress test is the most important clinical assessment for detecting medial collateral ligament injury. This test has high sensitivity (100%) and moderate specificity (75%) [30]. With the patient’s shoulder abducted and externally rotated, the examiner applies a constant valgus force while moving the elbow from full flexion to extension. A positive test elicits pain along the medial elbow, a sense of instability, and increased medial joint opening compared to the contralateral side. The painful arc typically occurs between 120° and 70° of flexion, with maximal discomfort often reported at 90°, suggesting this as the critical zone of peak valgus stress. Additionally, the Milking Maneuver test is performed with the elbow flexed >90°, shoulder abducted and externally rotated, and forearm in supination. A valgus stress is applied by pulling on the patient’s thumb. A positive test reproduces pain or instability at the medial elbow [31].

Imaging

Plain radiographs help exclude fractures, joint incongruity, or loose bodies, and can reveal calcifications or avulsion-type injuries [32]. MRI remains the gold standard for UCL evaluation, demonstrating signal changes within the ligament and detecting concomitant pathology [33]. Dynamic US provides additional functional assessment, particularly in partial-thickness injuries [34].

Conservative Treatment

UCL injuries in contact athletes differ from those seen in overhead throwing athletes, typically presenting as acute traumatic events rather than chronic overuse conditions. This distinction supports a more favorable response to nonoperative treatment for partial tears, and in select cases, even for complete tears, depending on factors such as tear location, degree of ligament retraction, and athlete-specific demands. A personalized management strategy is essential to optimize recovery and facilitate return to play [35]. Conservative management typically involves cessation of the aggravating activity, followed by its gradual reintroduction once pain subsides, with attention to correcting any underlying technical errors. Physical therapy should emphasize strengthening the periscapular muscles, rotator cuff, core muscles, and flexor-pronator mass to enhance elbow stability and reduce the risk of reinjury prior to starting a progressive throwing program. Additionally, adjunctive biological therapies like PRP show promise as adjuncts to enhance the effectiveness of conservative management for partial UCL injuries [36]. Rettig et al. proposed a two-phase rehabilitation protocol. Phase I focuses on rest, symptom control, and therapeutic modalities. Phase II introduces progressive strengthening and a structured return-to-throwing program [37].

The rehabilitation period typically spans three to six months, with return-to-play success rates between 42% and 50%. However, it is important to note that particular consideration was given to distinguishing between new and chronic partial tears, with chronic cases being more likely to proceed toward UCL reconstruction. Surgical intervention was typically considered if the athlete failed to show improvement after an initial six to eight weeks of conservative rehabilitation [31].

Surgical Treatment

Some conditions that require surgery are a complete tear or valgus instability, failed conservative treatment, high-demand overhead athlete, ulnar nerve symptoms of associated joint pathology, and need for faster or more reliable return to sport [2]. As time goes by, there are several reconstructive techniques, including the Jobe technique, the docking technique, and the hybrid screw technique.

The modified Jobe technique approach involves a muscle-splitting incision (typically through the flexor carpi ulnaris) to minimize soft tissue disruption, followed by the creation of bone tunnels in the ulna and humerus [38]. A palmaris longus tendon graft (autograft or allograft) is woven in a figure-eight pattern to restore ligament function. Anterior transposition of the ulnar nerve is often performed [39]. Ahmad and ElAttrache reported a 93% return-to-sport rate in professional athletes, establishing this technique as the gold standard [40].

The docking technique is a modification of the Jobe technique that uses a simplified humeral tunnel configuration and suture fixation for precise graft tensioning. This technique reduces bone tunnel diameter and minimizes cortical breach [39]. Rohrbough et al. reported a 92% return to prior or higher levels of performance within one year [41].

The hybrid screw technique combines docking on the humeral side with an interference screw on the ulnar side [42]. This allows for secure graft fixation with lower risk to periarticular structures, although care must be taken due to the small size of the ulnar footprint [39].

Savoie et al. introduced a technique using hamstring allografts, avoiding donor-site morbidity. In a series with standard arthroscopic follow-up, 93% achieved good to excellent results, with 98% of patients reporting satisfaction. Based on Conway-Jobe classification, outcomes were excellent in 80%, good in 13%, fair in 7%, and none rated as poor [43].

Cubital tunnel syndrome

**Figure 3:**
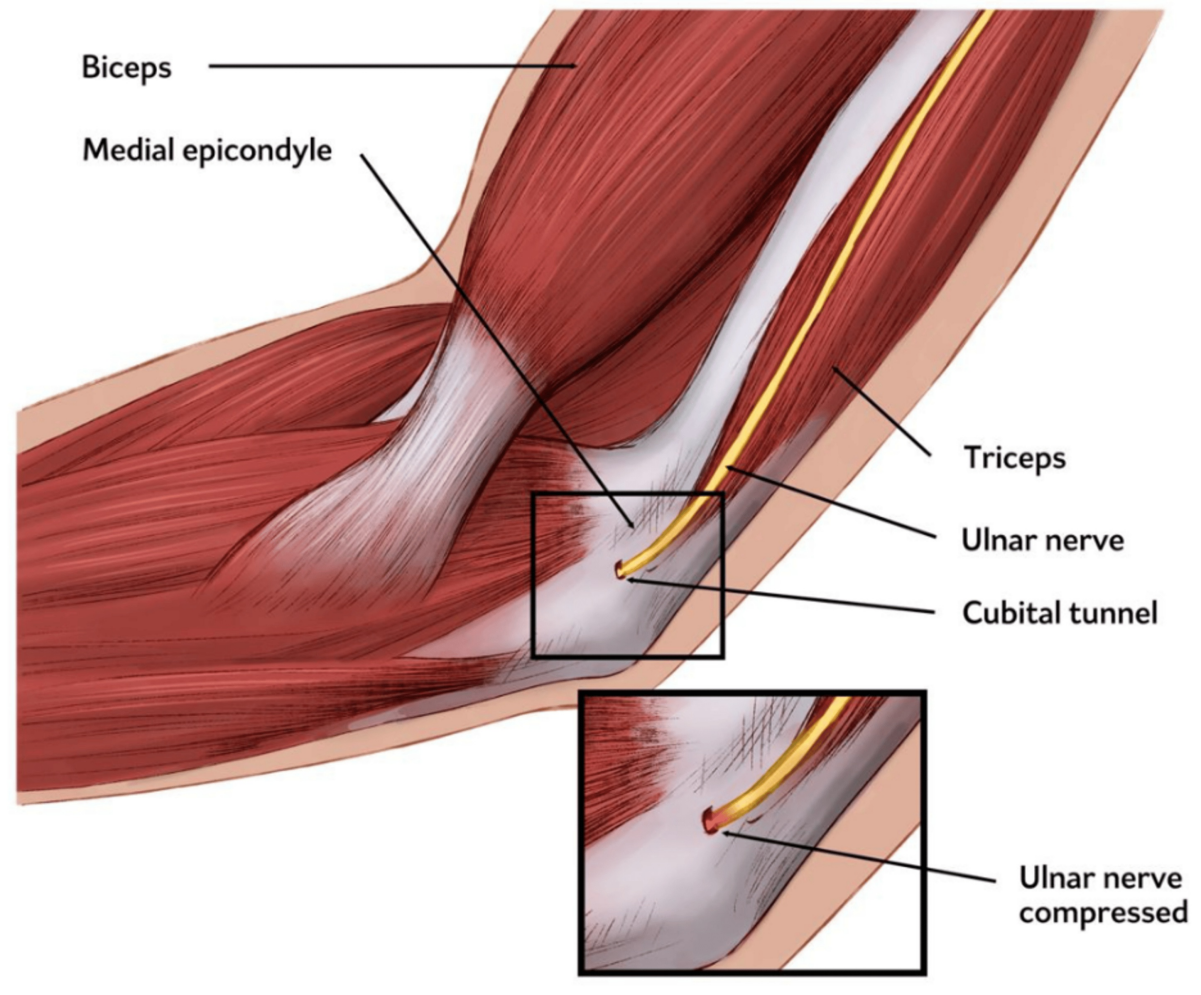
Illustration of cubital tunnel syndrome Image credit: The authors.

In addition to tendinous and ligamentous pathology, nerve compression syndromes such as cubital tunnel syndrome also contribute to medial elbow pain (Figure 3). It is the second most common upper-extremity compressive neuropathy after carpal tunnel syndrome [44]. Compression may result from both anatomical variations and extrinsic factors. Anatomical causes include thickening of the cubital tunnel retinaculum or the presence of accessory muscles, such as the anconeus epitrochlearis, which can exert compressive forces during elbow flexion [39]. Extrinsic causes include trauma, inflammatory arthropathies, space-occupying lesions (e.g., ganglion cysts, tumors), vascular anomalies, and osteoarthritic changes. Additionally, dynamic subluxation or dislocation of the ulnar nerve from its groove can lead to positional or intermittent compression. Elbow flexion tightens the cubital tunnel, increasing intraneural pressure and making the nerve particularly susceptible during repetitive flexion-extension activities [2].

Clinical Evaluation

A thorough patient history is essential in diagnosing cubital tunnel syndrome. Key symptoms typically include numbness and paresthesia, particularly in the ring and little fingers. Patients may also report pain that worsens with certain positions or repetitive activities involving elbow flexion. Hand weakness, especially affecting grip strength, may be noted. Clinicians should specifically inquire about difficulties with fine motor tasks, such as buttoning clothes, opening bottles, or typing, as these activities often reveal subtle impairments in hand function and precision pinch strength [45,46].

Cubital tunnel syndrome is primarily diagnosed clinically, with support from physical examination and confirmatory tests. Inspection and palpation will assess muscle atrophy, subluxation during elbow flexion, and any deformity or swelling. Common provocative maneuvers include Tinel’s sign, where tapping over the cubital tunnel elicits paresthesias in the ulnar distribution (sensitivity ~70%), and the elbow flexion test, which reproduces symptoms with sustained elbow flexion [43]. A newer method, the stretch-collapse test, involves stretching the skin over the nerve while the patient performs resisted shoulder external rotation. A brief loss of resistance indicates a positive result, likely due to allodynia from nerve compression [47]. Neurological testing can be done, which includes both sensory and motor assessments. Sensory evaluation involves two-point discrimination, Semmes-Weinstein monofilament testing, and vibration testing to detect subtle deficits. Motor assessment focuses on signs of intrinsic hand muscle weakness, including Froment’s sign, Wartenberg’s sign, and the cross-over test.

Imaging and Electrodiagnostics

Various imaging modalities may be employed, each serving a distinct purpose. Plain radiographs assess osseous abnormalities. US enables dynamic visualization, while MRI is useful in complex cases with suspected space-occupying lesions [39,48]. Electrodiagnostic studies remain the gold standard, localizing the site of compression and grading severity [48].

Conservative Treatment

Conservative management is the first-line approach for mild or early-stage disease and typically includes activity modification (e.g., avoiding prolonged elbow flexion beyond 45-70°), night splinting, nerve gliding exercises, and the use of elbow pads to prevent direct trauma [49]. Studies have reported symptom improvement in 60% to 95% of patients treated with non-operative measures. However, no single modality, such as splinting, exercises, or activity modification, has been shown to be clearly superior to the others [49,50]. Conservative management has been shown to yield favorable outcomes in mild cases, with success rates reaching up to 90%, and approximately 35% to 42% of patients achieving complete symptom resolution within six months [49,50].

Surgical Treatment

Surgical intervention is considered in cases where conservative treatment fails, when there is progressive motor weakness, moderate-to-severe compression demonstrated on electrodiagnostic testing, or in the presence of symptomatic ulnar nerve subluxation. Several surgical options are available, including simple in situ decompression, which relieves pressure without transposing the nerve, and medial epicondylectomy [51]. This surgical approach is associated with a shorter operative time (14 minutes versus 31 minutes) and a lower complication rate (10% compared to 31%) when compared to the anterior transposition technique. However, the majority of surgeons recommend either nerve transposition or medial epicondylectomy for patients with a hypermobile ulnar nerve [52]. Anterior transposition is another widely used technique and can be performed through different approaches. Subcutaneous transposition offers ease of access and has been associated with lower recurrence rates compared to in situ decompression. In contrast, intramuscular and submuscular transpositions are typically reserved for severe, recurrent, or revision cases. Meta-analyses have not demonstrated significant superiority of anterior transposition over in situ decompression in idiopathic, non-traumatic cases [53-56]. Nonetheless, transposition procedures may carry a higher risk of wound complications, and revision surgeries reported in up to 51% of cases are more frequently required due to perineural fibrosis at the medial intermuscular septum [51]. Surgical interventions demonstrate promising results, with return-to-activity rates varying by each technique. In situ decompression, the least invasive surgical option, has a return-to-activity rate of 75.7% but is associated with a greater risk of symptom recurrence. Subcutaneous transposition offers a higher success rate of 87.9%, while submuscular transposition is typically chosen for more severe cases or revision surgeries, and it may achieve rates as high as 95%. However, the selection of surgical technique depends on multiple factors, including the severity of symptoms, ulnar nerve stability, patient-specific anatomical considerations, and any prior surgical history [51,54,55,57].

Snapping medial triceps syndrome

Snapping medial triceps syndrome is a rare cause of medial elbow pain, often associated with dynamic ulnar nerve subluxation. It is typically seen in males performing repetitive upper-extremity loading (e.g., weightlifting, push-ups) [58]. The snapping phenomenon may result from a combination of anatomical and pathological factors, including accessory triceps heads or anomalous muscle insertion, triceps hypertrophy, hypoplasia of the medial epicondyle, cubitus varus deformity, post-traumatic changes or instability (e.g., posterolateral rotatory instability), and intra-articular adhesions, osteochondral defects, loose bodies, annular ligament injury, or synovial folds [59].

During elbow flexion, especially against resistance, the medial portion of the distal triceps may widen, potentially displacing the ulnar nerve from its groove. This can result in subluxation or even dislocation anterior to the medial epicondyle, often producing an audible or palpable “snap”. In some cases, the medial border of the triceps itself may subluxate with further elbow flexion, causing a second distinct snap [2].

Clinical Evaluation

**Figure 4:**
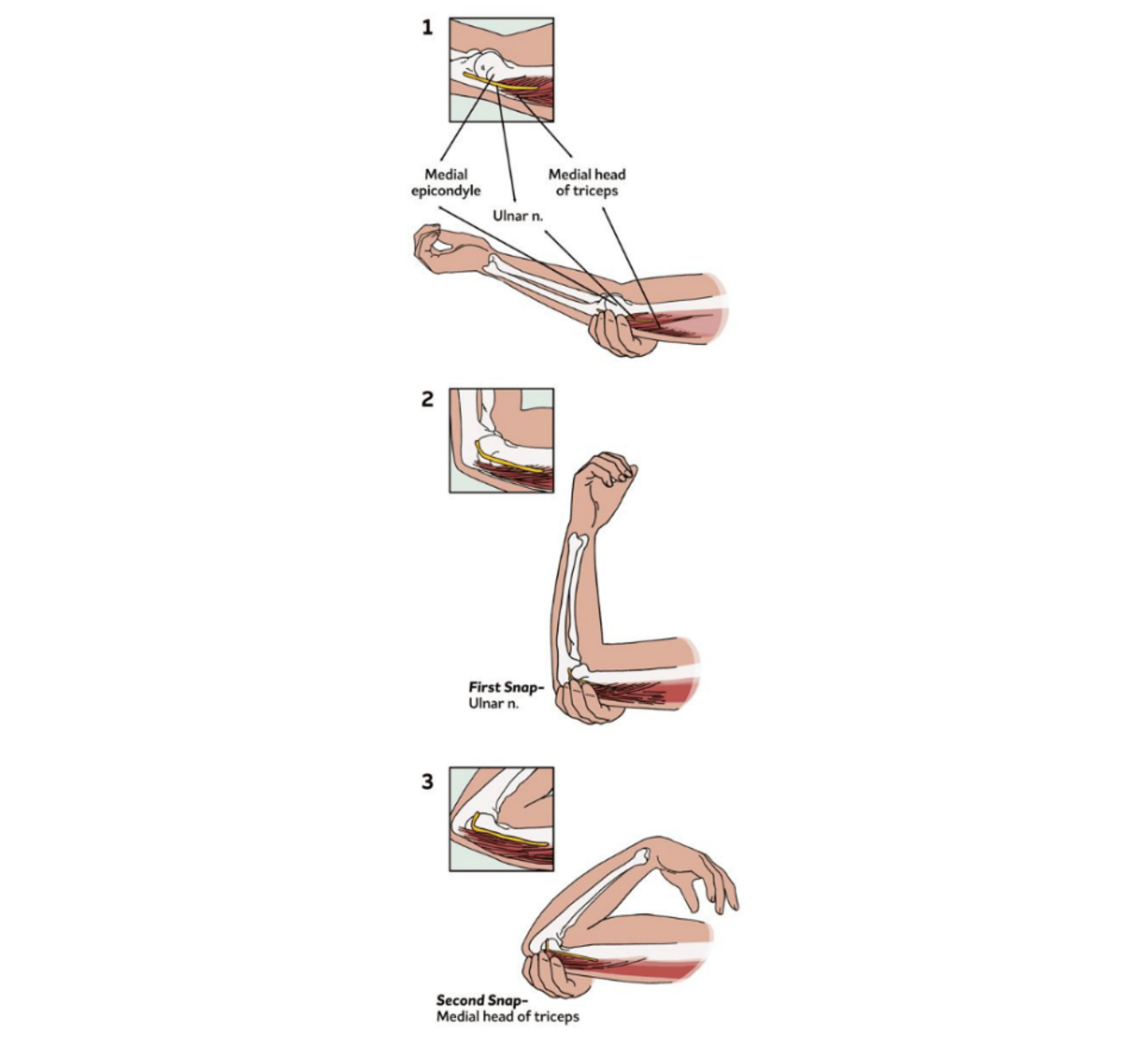
Physical examination findings (1) With the elbow fully extended, the ulnar nerve and the medial triceps are posterior to the medial epicondyle. (2) At approximately 90°, the ulnar nerve has dislocated over the medial epicondyle. (3) At approximately 115°, the medial triceps has dislocated over the medial epicondyle. Image credit: The authors.

The primary goal of clinical evaluation is to distinguish between ulnar nerve subluxation and medial triceps tendon subluxation. A first palpable snap occurring around 90° of elbow flexion often indicates ulnar nerve subluxation or dislocation. A second snap, typically felt between 110° and 120° of flexion, may suggest subluxation of the medial triceps tendon (Figure 4) [2,60]. Palpation during active elbow flexion-extension, especially against resistance, helps localize the source of snapping [60].

Imaging

Dynamic US is the preferred imaging modality due to its ability to visualize real-time displacement of the ulnar nerve and medial triceps. It is particularly effective even in asymptomatic patients or those with intermittent snapping, although results are highly operator-dependent [60,61]. MRI, particularly with the elbow in flexion, can reveal dislocation of the ulnar nerve or snapping of the medial head of the triceps and is useful in surgical planning or when prior interventions have failed [61].

Nonoperative Management

Initial treatment of snapping medial triceps syndrome is conservative and typically trialed for three to six months. It includes modifying activities by avoiding weightlifting, push-ups, and swimming, using anti-inflammatory medications, applying elbow orthosis positioned at about 70° of flexion, and wearing night braces to prevent prolonged elbow flexion [62].

Surgical Management

Surgical intervention is considered in cases with persistent or severe symptoms, particularly when conservative treatment fails. It is also indicated when there is associated cubital tunnel syndrome or evidence of structural instability requiring correction [63].

Surgical strategies are tailored to the specific structures involved. In case of ulnar nerve decompression, with or without anterior transposition, subcutaneous transposition is commonly used and effective in most cases, while submuscular transposition may be needed in revisions or when there is significant nerve mobility or scarring. Management of the medial triceps tendon may involve stabilization, lateral transposition of the snapping portion, or partial excision if it is hypertrophic or redundant. Intraoperative dynamic exploration is crucial for accurately identifying the underlying cause and preventing persistent postoperative symptoms [2]. When relevant, correction of associated deformities such as cubitus varus should also be considered [64]. Postoperative management includes placing the elbow in an orthosis at 90 degrees of flexion for the first two weeks. After this period, patients are permitted full active and passive ROM. However, forceful passive flexion and any exercises involving triceps contraction are restricted until three months post-surgery. For throwing athletes, physical therapy begins at two weeks, initially emphasizing ROM, followed by a gradual progression of activities aimed at achieving full return to play by three months [63].

Posteromedial impingement

Posteromedial impingement occurs mainly in overhead athletes due to repetitive valgus torque and shear stress during terminal elbow extension [65]. It is frequently associated with poor throwing mechanics, inadequate dynamic muscular control, and insufficiency of the UCL. Chronic valgus stress, particularly in the presence of UCL laxity, leads to increased posterior contact pressures, which predispose the elbow to structural changes such as posteromedial olecranon osteophyte formation, chondromalacia of the olecranon fossa or posteromedial trochlea, and the development of intra-articular loose bodies [65,66]. These pathological changes can result in posteromedial elbow pain and mechanical symptoms, including catching or locking, as well as progressive valgus instability. If not appropriately managed, ongoing valgus overload may contribute to lateral compartment degeneration, manifesting as radiocapitellar synovitis and osteochondral injury [65].

Clinical Evaluation

Diagnostic maneuvers are employed to reproduce impingement symptoms and evaluate valgus instability. The extension impingement test involves repeated terminal elbow extension, which elicits posteromedial discomfort indicative of impingement. The arm bar test simulates forced elbow extension against resistance applied by the examiner, aiming to reproduce the patient’s pain. Valgus stress testing, including the moving valgus stress test and the milking maneuver, is used to assess the integrity of the UCL [65]. Additionally, assessment of the flexor-pronator mass and the ulnar nerve is crucial, as tenderness over the pronator mass and clinical signs of neuritis or ulnar nerve subluxation are often associated with medial elbow pathologies [67].

Imaging

Radiographs may reveal olecranon osteophytes or loose bodies; CT is useful for bony assessment. MRI provides a detailed assessment of soft tissue pathology, including UCL injury and chondral lesions, while dynamic US can evaluate valgus instability [65,67,68].

Conservative Treatment

Initial management is typically conservative and includes activity modification, particularly the avoidance of valgus-loading activities. Physical therapy plays a central role, with an emphasis on stretching, strengthening, and optimizing throwing mechanics. Pharmacological interventions, such as NSAIDs and corticosteroid injections, may be used to reduce inflammation and alleviate symptoms [65]. In selected cases, orthobiologic treatments such as PRP injections may be considered as adjunctive therapy. A gradual return-to-throwing program, accompanied by thorough biomechanical assessment and correction, is essential to reduce the risk of recurrence and ensure safe resumption of athletic activity [65,67].

Surgical Treatment

Surgical intervention is indicated in patients who continue to experience symptoms despite adequate conservative management, particularly when symptomatic osteophytes or loose bodies are present, or when mechanical symptoms interfere with daily function or athletic performance [33]. Surgical options include arthroscopic resection of posteromedial olecranon osteophytes and, when necessary, limited incision arthrotomy to allow open access for debridement or removal of large intra-articular loose bodies. When surgical management is combined with correction of underlying instability and other mechanical contributors, outcomes are generally favorable, with most athletes able to return to their previous levels of activity [33,65].

**Figure 5:**
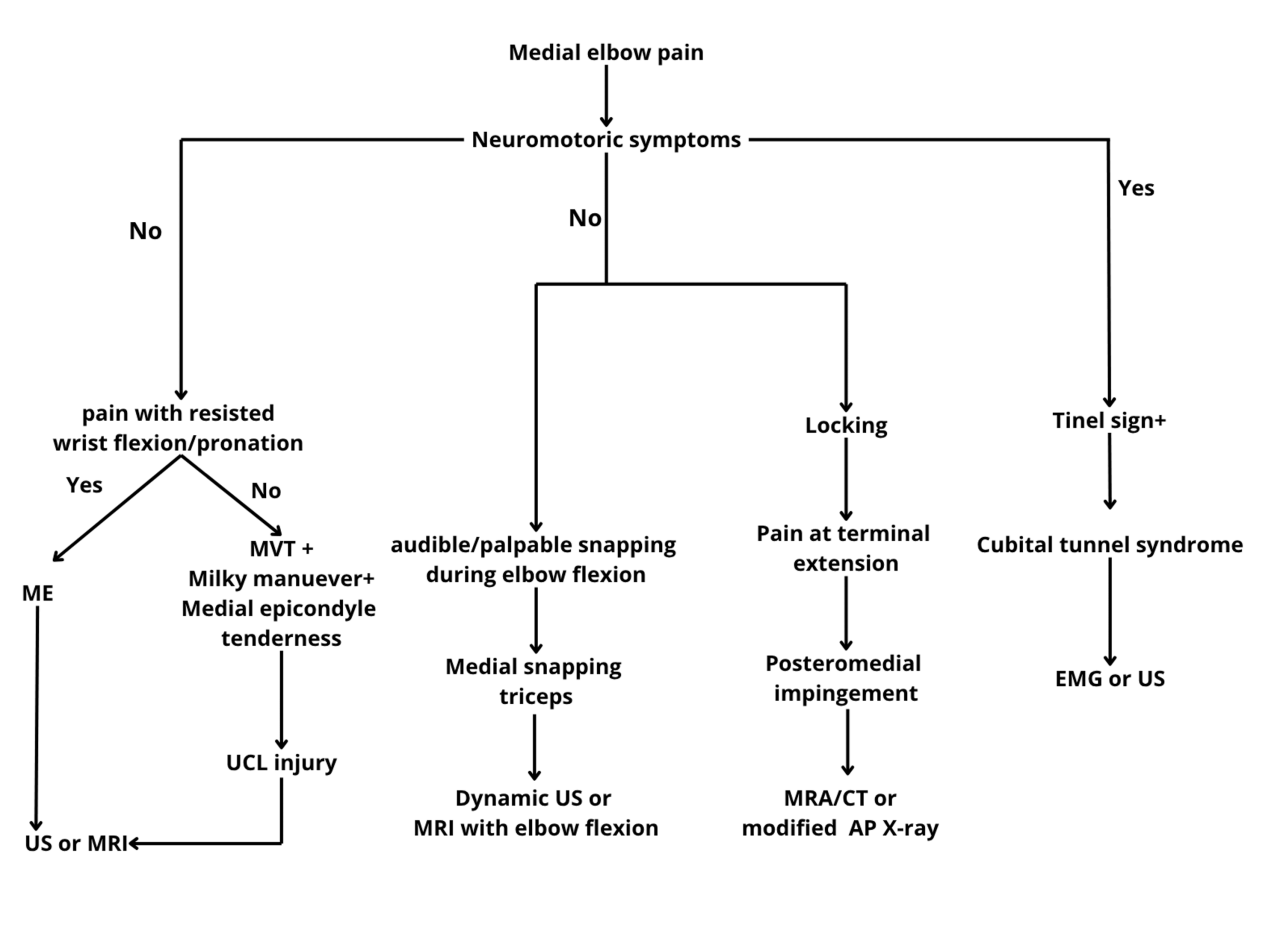
Diagnostic algorithm for non-traumatic medial-sided elbow pain ME: medial epicondylitis; MVT: moving valgus test; UCL: ulnar collateral ligament; US: ultrasonography; MRI: magnetic resonance imaging; MRA: magnetic resonance arthrography; EMG: electromyography; AP: anteroposterior Image credit: The authors.

A detailed summary highlighting the key aspects and essential findings from the comprehensive review on non-traumatic medial elbow pain is presented in Figure 5 and Table 1.

**Table 1:** Summary of management and outcomes of non-traumatic medial elbow pain UCL: ulnar collateral ligament; US: ultrasonography; MRI: magnetic resonance imaging; CT: computed tomography; AP: anteroposterior; PT: physical training; NSAIDs: non-steroidal anti-inflammatory drugs; PRP: platelet-rich plasma

Condition	Key Features	Diagnostics	Nonoperative Management	Surgical Options	Outcomes
Medial epicondylitis	Pain over the medial epicondyle; worsens with resisted wrist flexion/pronation	Clinical tests (e.g., Polk); US/MRI for tendon changes	PT, NSAIDs, bracing, injections (PRP, corticosteroids)	Open/arthroscopic debridement, decortication, and ulnar nerve transposition if needed	85% return to activity; some persistent pain with heavy use
UCL injury	Medial elbow pain during throwing; instability with valgus stress	Moving valgus stress test, milking maneuver; MRI, CT arthrography	Rest, rehab protocol, activity modification	Jobe, docking, hybrid screw, or allograft reconstruction techniques	92-93% return to sport with surgical repair
Cubital tunnel syndrome	Numbness/tingling in ulnar digits; weakness in hand grip	Tinel's sign, nerve conduction studies, MRI/US	Splinting, nerve gliding, and activity modification	Simple decompression, medial epicondylectomy, and anterior transposition	Up to 95% success post-transposition; recurrence is possible with in situ
Snapping medial triceps	Audible/palpable snap during elbow flexion, especially under load	Dynamic US, MRI with elbow flexion	Activity modification, NSAIDs, elbow orthosis	Ulnar nerve decompression ± transposition, triceps resection, or stabilization	Good outcomes when structural causes are addressed; most return to sport
Posteromedial impingement	Pain at terminal extension; mechanical symptoms (catching, locking)	Extension impingement test, MRI/CT, modified AP X-ray	PT, NSAIDs, orthobiologics, throwing program	Arthroscopic debridement, limited open arthrotomy	Favorable return to activity post-debridement and correction

Future directions

To improve the effectiveness and precision of managing medial-sided elbow pain, several key areas should be prioritized in future research. High-quality prospective studies are essential to compare conservative and surgical interventions across various underlying pathologies. Direct comparisons between surgical techniques, such as simple decompression versus anterior transposition, using standardized outcome measures, are also needed. Moreover, conducting larger multicenter trials will enhance the generalizability of findings and increase statistical power. Additionally, establishing diagnostic thresholds that integrate clinical, neurophysiological, and imaging criteria is crucial for guiding the selection of appropriate treatment. Furthermore, long-term follow-up studies evaluating functional outcomes, recurrence rates, and return to sport or activity are necessary. These research directions are expected to help bridge current evidence gaps, refine treatment algorithms, and promote evidence-based, patient-centered care in the management of medial elbow pathologies.

## Conclusions

Non-traumatic medial elbow pain can result from a variety of underlying conditions. Accurate diagnosis relies on a thorough patient history, a comprehensive physical examination, and appropriate imaging studies. While the majority of cases respond well to conservative management, evaluation of ulnar nerve involvement is crucial, as such cases often require surgical intervention.

Despite advancements in diagnostic techniques, significant gaps remain in guiding optimal treatment strategies. There is no consensus on when to escalate care from conservative to surgical management, particularly for conditions such as idiopathic ulnar neuropathy and cubital tunnel syndrome. Clinical decision-making is often based on limited or low-quality evidence, underscoring the need for stronger data to support therapeutic choices.
